# The *Arabidopsis thaliana* F-box gene *HAWAIIAN SKIRT* is a new player in the microRNA pathway

**DOI:** 10.1371/journal.pone.0189788

**Published:** 2017-12-15

**Authors:** Xuebin Zhang, Dasuni Jayaweera, Janny L. Peters, Judit Szecsi, Mohammed Bendahmane, Jeremy A. Roberts, Zinnia H. González-Carranza

**Affiliations:** 1 Plant and Crop Sciences Division, School of Biosciences, University of Nottingham, Sutton Bonington Campus, Loughborough, Leicestershire, United Kingdom; 2 Department of Molecular Plant Physiology, Institute for Water and Wetland Research, Radboud University Nijmegen, Nijmegen, The Netherlands; 3 Laboratoire Reproduction et Développement des Plantes, Univ Lyon, ENS de Lyon, Université Claude Bernard Lyon 1, CNRS, INRA, Lyon, France; Kunming University of Science and Technology, CHINA

## Abstract

In Arabidopsis, the F-box *HAWAIIAN SKIRT* (*HWS*) protein is important for organ growth. Loss of function of *HWS* exhibits pleiotropic phenotypes including sepal fusion. To dissect the *HWS* role, we EMS-mutagenized *hws-1* seeds and screened for mutations that suppress *hws-1* associated phenotypes. We identified *shs-2* and *shs-3* (*s**uppressor of* *h**w**s**-2* and *3*) mutants in which the sepal fusion phenotype of *hws-1* was suppressed. *shs-2* and *shs-3* (renamed *hst-23/hws-1* and *hst-24/hws-1*) carry transition mutations that result in premature terminations in the plant homolog of Exportin-5 *HASTY* (*HST*), known to be important in miRNA biogenesis, function and transport. Genetic crosses between *hws-1* and mutant lines for genes in the miRNA pathway also suppress the phenotypes associated with *HWS* loss of function, corroborating epistatic relations between the miRNA pathway genes and *HWS*. In agreement with these data, accumulation of miRNA is modified in *HWS* loss or gain of function mutants. Our data propose *HWS* as a new player in the miRNA pathway, important for plant growth.

## Introduction

Selective degradation of proteins is carried out via the ubiquitin-proteasome pathway which is fundamental for many cellular processes, including development, hormonal signalling, abiotic stress and immunity in plants [1, 2]. The abundance of key brakes and/or accelerators that control these processes is regulated by the 26S proteasome using complex mechanisms to avoid destruction of crucial proteins and the release of partially degraded polypeptides [2, 3]. E1, E2 and E3 enzymes sequentially attach the small soluble protein ubiquitin to the proteins destined for degradation [1, 4]. The E3 ligase enzyme provides the specificity when it binds to the target substrate and the activated ubiquitin-E2 complex; the polyubiquitinated substrates are then degraded by the 26S proteasome [1, 5]. The SCF E3 ligase is composed of four subunits: S-phase-kinase-associated protein-1 (Skp1), Cullin (Cul1), RING-finger protein (Rbx1/Roc1) and F-box protein (SCF complex) [3, 6].

In Arabidopsis it has been shown that 21 SKP (or ASK- ARABIDOPSIS SKP1 RELATED) genes are expressed [7] while 692 F-box genes proteins have been identified in the genome [8]. The targets for degradation for a few of the F-box proteins have been identified, such as the receptor of auxin TRANSPORT INHIBITOR RESPONSE 1 (TIR) [9, 10]; the auxin response regulators ABF1, 2 and 3 [9]; CORONATINE INSENSITIVE 1 (COI1) that targets ZIM-domain (JAZ) proteins for degradation in response to JA perception [11]; AtSKIP18 and AtSKIP31 that target for degradation 14-3-3 proteins [12] and ZEITLUPE (ZTL) that targets for degradation CRYPTOCHROME-INTERACTING basic helix–loop–helix 1 (CIB1) [13]. Even though a considerable amount of information related to their function has been reported, the targets for many F-box proteins remain elusive.

We have identified that the Arabidopsis F-box protein *HAWAIIAN SKIRT* (*HWS*) has a key role in regulating plant growth and flower development, cell proliferation and control of size and floral organ number [14]. The *hws-1* mutant is pleiotropic and its most conspicuous phenotype is the sepal fusion of flowers precluding floral organ shedding [15]. This phenotype is similar to that of the double mutant *cuc1*/*cuc2* [*CUP-SHAPED COTYLEDON 1* (*CUC1*) and 2 (*CUC2*)] [16] and to that of the *Pro*_*35*_:*164B* ectopic lines for the microRNA gene *MIR164B* [17, 18]. Recently we demonstrated that HWS controls floral organ number by regulating transcript accumulation levels of the *MIR164*. Very recently, we showed that, *HWS* indirectly regulates accumulation of *CUC1* and *CUC2* genes mRNA [14].

Furthermore, the leaf and floral phenotypes in HWS overexpressing plants (*Pro*_*35*_:*HWS*) are remarkably similar to mutants involved in the miRNA pathway, including leaf serration [15]. However, no direct link between HWS and miRNA biogenesis, nuclear export or function of miRNAs has been described.

MicroRNAs (miRNAs) or small RNAs are sequence-specific guides of 19–24 nucleotides that repress the expression of their target genes [1, 19]. In plants, miRNAs were shown to be involved in vegetative and reproductive developmental processes, to be directly or indirectly associated with various signalling pathways, such as auxin, CK, ABA hormonal pathways, among others [17–18, 20–28].

The complexity of miRNA biogenesis has become apparent in recent years (for reviews see 29–33]. In plants, miRNAs originate from a primary miRNA transcript (pri-miRNA) transcribed by RNA polymerase II, the miRNAs form foldback structures by imperfect pairing [19, 32, 34]. DAWDLE (DDL), a FHA domain-containing protein in Arabidopsis, interacts with the endoribonuclease helicase with RNase motif DICER-LIKE1 (DCL1) to facilitate access or recognition of pri-miRNAs [35]. STABLILIZED1 (STA1), a pre-mRNA processing factor 6 homolog modulates *DCL1* transcription levels [36]. In the D-body, a complex that includes the C2H2-zinc finger protein SERRATE (SE), the double-stranded RNA-binding protein HYPONASTIC LEAVES-1 (HYL-1), DCL-1 and a nuclear cap-binding complex (CBC), process the pri-mRNA to generate a pre-miRNA [37– 41]. PROTEIN PHOSPHATASE 4 (PP4), SUPPRESOR OF MEK1 (SMEK1) [42], REGULATOR OF CBF GENE EXPRESSION (RCF3) and C-TERMINAL DOMAIN PHOSPHATASE-LIKE1 AND 2 (CPL1 and CPL2) control the phosphorylation status of HYL-1 to promote miRNA biogenesis [43]. The mature sRNA duplexes (miRNA/miRNA*) are either retained in the nucleus or exported to the cytoplasm once they are stabilized by the S-adenosyl methionine dependent methyltransferase HUA ENHANCER 1 (HEN-1) [44–46], which protects them from degradation by the SMALL RNA DEGRADING NUCLEASE (SDN) exonucleases [47]. HASTY (HST), the plant homolog of Exportin-5 (Exp5), is involved in biogenesis or stability of some miRNAs and in transporting a yet to be identified component in the miRNA pathway [48]. The guide miRNA strand is merged into ARGONAUTE (AGO) proteins which carry out the post transcriptional gene silencing reactions (PTGS) [48–49].

In animals, regulation of miRNA biogenesis occurs at multiple levels. It occurs at the transcriptional level, during processing by Drosha (in the nucleus) and Dicer (in the cytoplasm), as well as by RNA editing, RNA methylation, urydylation, adenylation, AGO loading, RNA decay and by non-canonical pathways for miRNA biogenesis [50–51]. Although a vast amount of information has emerged relating to the biogenesis of miRNAs in plants, the mechanisms that modulate miRNAs and their generators in the canonical pathway, and/or the presence of non-canonical pathways are yet to be elucidated.

Here, we describe the identification and mapping of two *hws-1* suppressor mutants (*hst-23* and *hst-24*) in which the *hws-1* sepal fusion phenotype is suppressed. These mutants are new mutant alleles of *HASTY* known to be involved in biogenesis or stability of some miRNAs and transporting of an unidentified component in the miRNA pathway. We demonstrate that mutation of *HST* as well as mutations of other genes in the miRNA biogenesis pathway and function are able to suppress *hws* phenotypes and vice versa. In agreement with these findings, the levels of miR163 and miR164 mature miRNAs in floral tissues are modified in lines that exhibit a loss or gain of function for HWS. The data support the hypothesis that *HWS* is a previously unidentified regulator of the miRNA pathway.

## Material and methods

### Plant material

Seeds from Col-0 (N60000), *ddl-2* (N6933), *se-1* (N3257), *hyl-1* (N3081), *dcl1-9* (N3828), *hen1-5* (N549197), *hst-1* (N3810) and *ago1-37* (N16278) were obtained from the Nottingham Arabidopsis Stock Centre. Homozygous lines were identified, when appropriate, before crossing them to *hws-1* or *hws-2* as described in [52]. The *hws-1* allele has a 28 bp deletion and has been isolated from a neutron fast bombardment mutagenized population, whereas the *hws-2* allele has two T-DNA insertions inserted in opposite directions 475 and 491 bp downstream the ATG [15]. All lines were grown in a growth room supplemented with fluorescent lights (200 μmol m^-2^s^-1^: Polulox XK 58W G-E 93331). The *hws-1* EMS populations grew in a greenhouse, temperature 23±2°C and photoperiod 16h light/8h darkness. All plants grew in plastic pots containing Levington M3 (The Scotts Company).

The *hws-1* EMS mutagenized seeds were generated, screened and confirmed to be true suppressors by using specific primers to detect *hws-1* mutation (S1 Table).

### Map-based cloning

To map the *shs-2* mutation, a F_2_ population was generated by selfing the F1 progeny from a cross between *shs-2/hws-1* (*hst-24/hws-1*) and *hws-5* (*ffo1*). DNA was extracted from about 120 F_2_ plants displaying a suppression of the sepal fusion phenotype of *hws-1* (Sigma-Aldrich, GeneElute^™^ Plant Genomic DNA Miniprep Kit).

To identify the chromosome containing the *shs-2* mutation, an AFLP-based genome-wide mapping strategy [53] was used on a subset of 40 DNA samples. Further mapping with all samples was performed with InDels [54]. For fine mapping, an additional 600 F2 plants were used. Once the region was narrowed down to a 59.4 Kb, candidate genes in the region were identified and a 6.927 Kb region of the *HST* gene was sequenced. A similar genomic region was amplified from the *shs-3/hws-1* line for sequencing. Allelism tests between *shs-2/hws-1* and *shs-3/hws-1* were carried out by reciprocal crossing between the mutants. Primers used for mapping and sequencing are summarized in S1 Table.

### Phenotypic analyses

The sepals and petals from twenty-five flowers (from six plants) from Col-0, *hws-1*, *hst-24/hws-1* and *hst-24* in Col-0 were carefully dissected, counted and photographed. Mature siliques and leaves dissected from 22 day-old plants from these lines were also recorded. Siliques from individual mutants and crosses between *hws-1*, *hws-2*, *ddl-2*, *se-1*, *hyl-1*, *dcl1-9*, *hen1-5*, *hst-1* and *ago1-37*, were recorded following the same procedure.

All data obtained were used to perform statistical analyses and to create graphics. Regression analyses and ANOVA using generalized linear models were performed using GenStat 17.1. Graphics were created using Microsoft Excel 2016 and annotated using Adobe Photoshop 7.0.1.

### miRNA Northern blots

Mature miRNAs were detected using the protocol described by [55]: total RNA was isolated from a cluster of buds and young flowers (up to stage 12, [56]) from Col-0, *hws-1*, and *Pro*_*35*_:*HWS* lines using TRIzol reagent (Life Technologies). Ten μg of total RNA from each line were used for northern hybridisation. Antisense probes were constructed using *mir*Vana^™^ miRNA Probe Construction kit (Ambion) and radio labelled with γATP^32^P. Sequence information of probes is included in S1 Table.

### Yeast two-hybrid assay

ProQuest^™^ yeast Two-hybrid system (Invitrogen) was used to study protein-protein interaction. The full length *HWS* coding region was cloned into pDEST32 and used to screen a stamen-specific tissue cDNA library [57]. Positive clones for Histidine bigger than 1mm in diameter were isolated and subjected to X-gal filter assays following manufacturer’s instructions (Invitrogen). Plasmid DNA was isolated from selected individual clones, and then sequenced to identify the corresponding genes. To confirm the interaction, X-gal assays were repeated with the isolated clones.

### Accession numbers

Sequence data from genes in this article can be found in the Arabidopsis Genome initiative or GenBank/EMBL databases under the following accession numbers: *HWS*, *At3g61590*; *HST*, *At3g05040; DDL*, *AT3G20550*; *SE*, *AT2G27100; HYL-1*, *AT1G09700; DCL-1*, *AT1G01040; HEN-1*, *AT4G20910; AGO-1*, *AT1G48410*.

## Results

### The mutants *shs-2* and *shs-3* are novel alleles of *HASTY* and suppress the sepal fusion phenotype of *hws-1*

**Fig 1:**
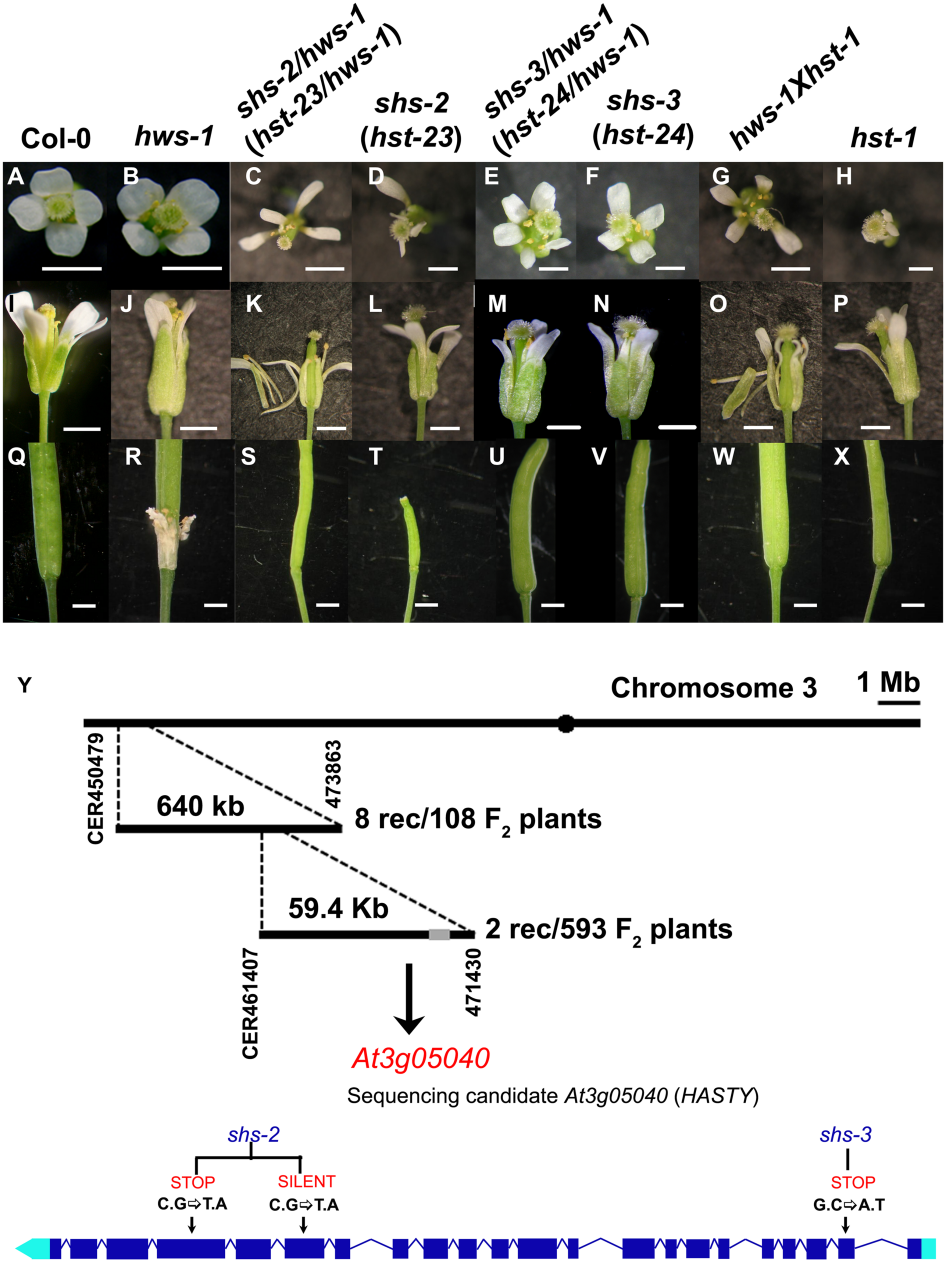
The *shs-2* and *shs-3* mutants are alleles of *HST*. (**A-H**), Aerial and (**I-P**), lateral views of flowers at stage 15a; and (**Q-X**), lateral view of mature green siliques from wild type in Col-0, *hws-1*, *shs-2/hws-1* (*hst-23/hws-1*), *shs-2* (*hst-23)*, *shs-3/hws-1* (*hst-24/hws-1*), *shs-3* (*hst-24)*, *hws-1xhst-1*, *hst-1*. Bars = 1mm. (**Y**), Mapping strategy used to identify the *hst-23 and hst-24* mutations. Structure of the gene and location of the transition substitution (C.G→T.A) at positions 4.587 Kb and 5.517 Kb in *hst-23* and (G.C→A.T) at 0.583 Kb in *hst-24* from the ATG are included, intragenic regions are represented by thin lines and exons by dark boxes.

To identify the substrate for the F-box *HAWAIIAN SKIRT* protein from Arabidopsis, we performed a suppressor screen by EMS-mutagenizing the *hws-1* mutant in a Columbia-0 (Col-0) background. Screening of 308 individuals from 43 M2 populations resulted in the identification of two suppressor lines *shs-2*/*hws-1* (*s**uppressor of* *h**ws-**2*) and *shs-3*/*hws-1* (*s**uppressor of* *h**ws-**3*) that displayed no sepal fusion, suggesting suppression of the *hws-1* phenotype (Fig 1I, 1J, 1K, 1M, 1Q, 1R, 1S and 1U). Reciprocal crosses between *shs-2*/*hws-1* and *shs-3*/*hws-1* yielded F1 individuals that displayed the same phenotype as the parents and restored the sepal fusion phenotype of *hws-1* (S1 Fig) demonstrating that these suppressor mutations are allelic. The suppressor *shs-2*/*hws-1* (in Col-0) was crossed to *hws-5* (*ffo-1*, Landsberg *erecta*, L*er* background) to generate a mapping population. The F1 individuals from this cross showed the sepal fusion phenotype suggesting that the mutant is recessive. The F2 population was then used for gene mapping. The *shs-2* mutation was located in a 59.4 Kb region at the top of chromosome 3 (Fig 1Y). This region contains 19 genes, including *At3g05040* (*HASTY-HST*), a gene known to be involved in the export of mature miRNA molecules from the nucleus to the cytoplasm [48–49]. Analyses of the genomic region containing the *HST* gene in *shs-2/hws-1* identified two transition mutations at positions 4.587 Kb and 5.517 Kb downstream from the ATG in *shs-2/hws-1* line, resulting in a silent (ATC→ ATT ~Ile) and a premature termination (CAG→ TAG; Gln →amber stop codon), respectively. In the *shs-3/hws-1* line a transition mutation was located 0.583 Kb downstream of the ATG, introducing an earlier termination (GTG→GTA; Val→amber stop codon; Fig 1Y). Consequently, the *shs-2* and *shs-3* mutants were renamed *hst-23* and *hst-24*. These mutations generate truncated versions of HST of 924 and 57 amino acids respectively, compared to the wild type HST protein consisting of 1202 aa. The double mutants *hst-23/hws-1* and *hst-24/hws-1* were back-crossed with Col-0 to obtain *hst-23* and *hst-24* single mutants for subsequent analyses (Fig 1D, 1F, 1L, 1N, 1T and 1V). The F2 progenies displayed a segregation ratio 3:1 confirming that these are single, recessive nuclear mutations. The *hst-23* allele displayed relatively more severe floral and vegetative phenotypes compared to *hst-24* allele (Fig 1 and S1 Fig).

To confirm that mutation of *HST* is responsible for the suppression of *hws* phenotype, we crossed *hws-1* with *hst-1*, an independent mutant that harbours a mutation in the *HST* coding region that generates a truncated protein of 521 amino acids with the last 18 aa differing from the wild type protein [58]. As shown in Fig 1G, 1O and 1W, flowers from F2 individuals displayed no sepal fusion, thus corroborating that mutation in *HST* is able to suppress the phenotype of *hws-1*. Taken together these data demonstrate that mutations in *HST* suppress the *hws* phenotype, thus suggesting a putative role of HWS function in miRNA transport pathway.

### *HWS* has a role in the miRNA pathway

*HST* is the Arabidopsis orthologue of Exp-5 from mammals, a protein involved in small RNAs export from the nucleus to the cytoplasm [48]. We previously showed that overexpression of HWS (*Pro*_*35*_:*HWS*) leads to phenotypes resembling those of mutants in miRNA pathway. This knowledge together with the fact that the *HWS* loss of function phenotype is suppressed by mutation in *HST*, prompted us to address if the *HWS* plays a role in miRNA biogenesis and function.

The *hws-1* and *hws-2* mutants [15] were crossed with lines mutated in genes known to act in the miRNA biogenesis pathway, and function, including *ddl-2*, *se-1*, *hyl-1*, *dcl1-9*, *hen1-5*, *hst-1* and *ago1-37*. Mutations in these genes are known to affect floral and vegetative development, including delayed growth, reduced fertility, defects in root, shoot and flower morphology, highly serrated leaves, severe leaf hyponasty, curling up of leaves and extra sepals and petals [35, 37– 41, 59–60].

**Fig 2:**
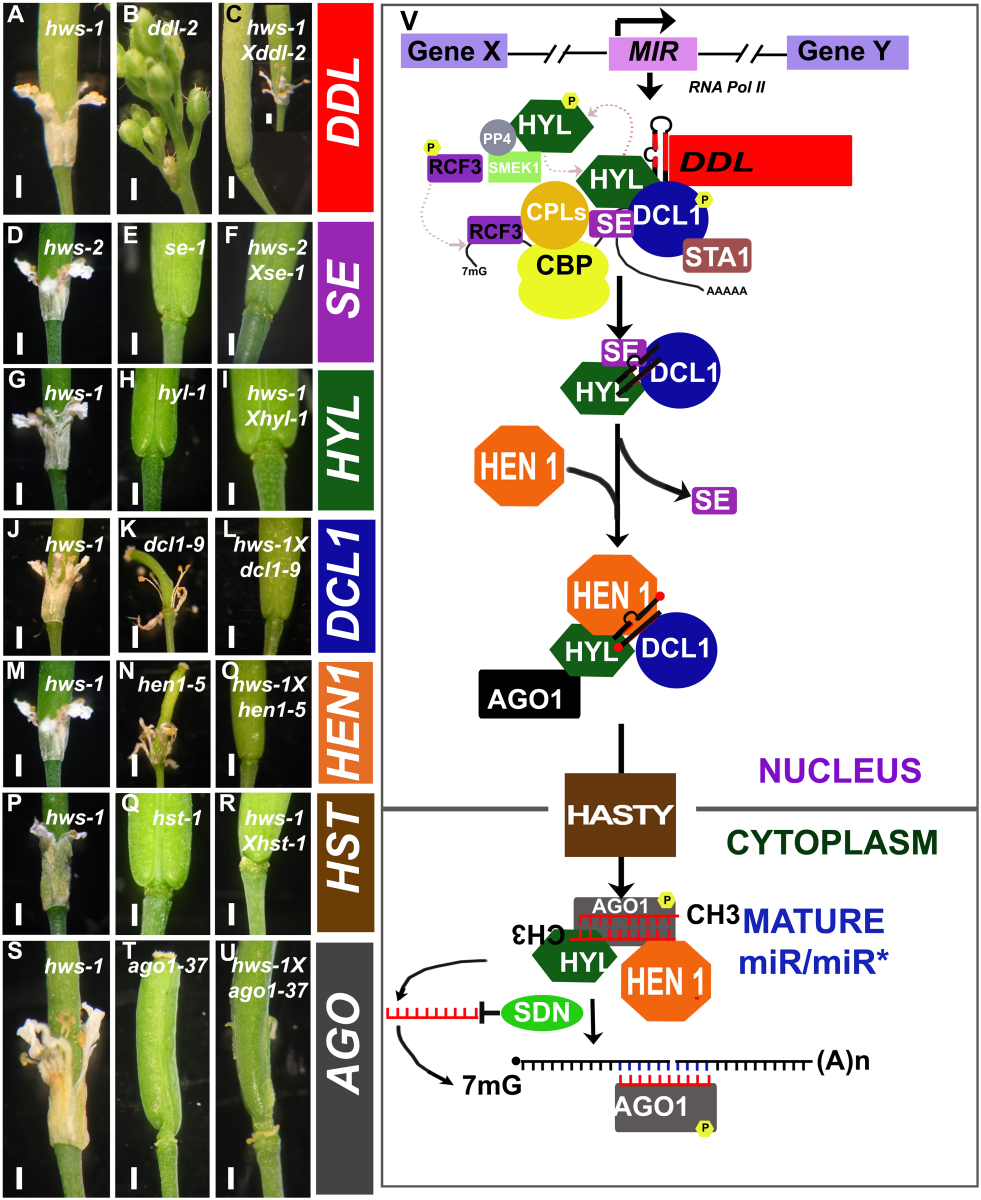
miRNA pathway and co-suppression between *hws-1* and miRNA pathway mutants. Single (**A, G, J, M, P, S**) *hws-1*, (**D**) *hws-2*, (**B**) *ddl-2*, (**E**) *se-1*, (**H**) *hyl-1*, (**K**) *dcl1-9*, (**N**) *hen1-5*, (**Q**) *hst-1*, (**T**) *ago1-37*, and double (**C**) *hws-1Xddl-2*, (**F**) *hws-1Xse-1*, (**I**) *hws-1Xhyl-1*, (**L**) *hws-1Xdcl1-9*, (**O**) *hws-1Xhen1-5*, (**R**) *hws-1Xhst-1*, (**U**) *hws-1Xago1-37*, mutants showing co-suppression of phenotypes. Bars = 1mm. The (**V**) miRNA pathway (modified from [32, 36, 61]) has been included for reference.

F2 plants were isolated and the double mutants identified by PCR. The genetic interactions showed that all tested miRNA biogenesis and function pathway mutants, were able to suppress the sepal fusion phenotype in the *hws-1* and *hws-2* independent mutants (Fig 2A, 2B, 2C, 2D, 2E, 2F, 2G, 2H, 2I, 2J, 2K, 2L, 2M, 2N, 2O, 2P, 2Q, 2R, 2S, 2T and 2U) the *hws-2* allele harbour two T-DNAs inserted in opposite directions 465 and 491 bp downstream the ATG of *HWS* [15]. Interestingly, the *hws* mutants were also able to suppress the phenotypes of these mutants in some instances. It is particularly noticeable that the *hws* mutant was able to suppress the delayed or arrested development from siliques of the mutants *ddl-2* (Fig 2A, 2B and 2C), *dcl1-9* (Fig 2J, 2K and 2L) and *hen1-5* (Fig 2M, 2N and 2O). It should be noted that in older plants, towards the end of the production of siliques, the reciprocal suppression of phenotypes between *hws* and the biogenesis pathways mutants was less apparent (data shown for *hws-1/ddl-2*; Fig 2C). These data support the proposal that *HWS* is an important regulator in the miRNA pathway.

**Fig 3:**
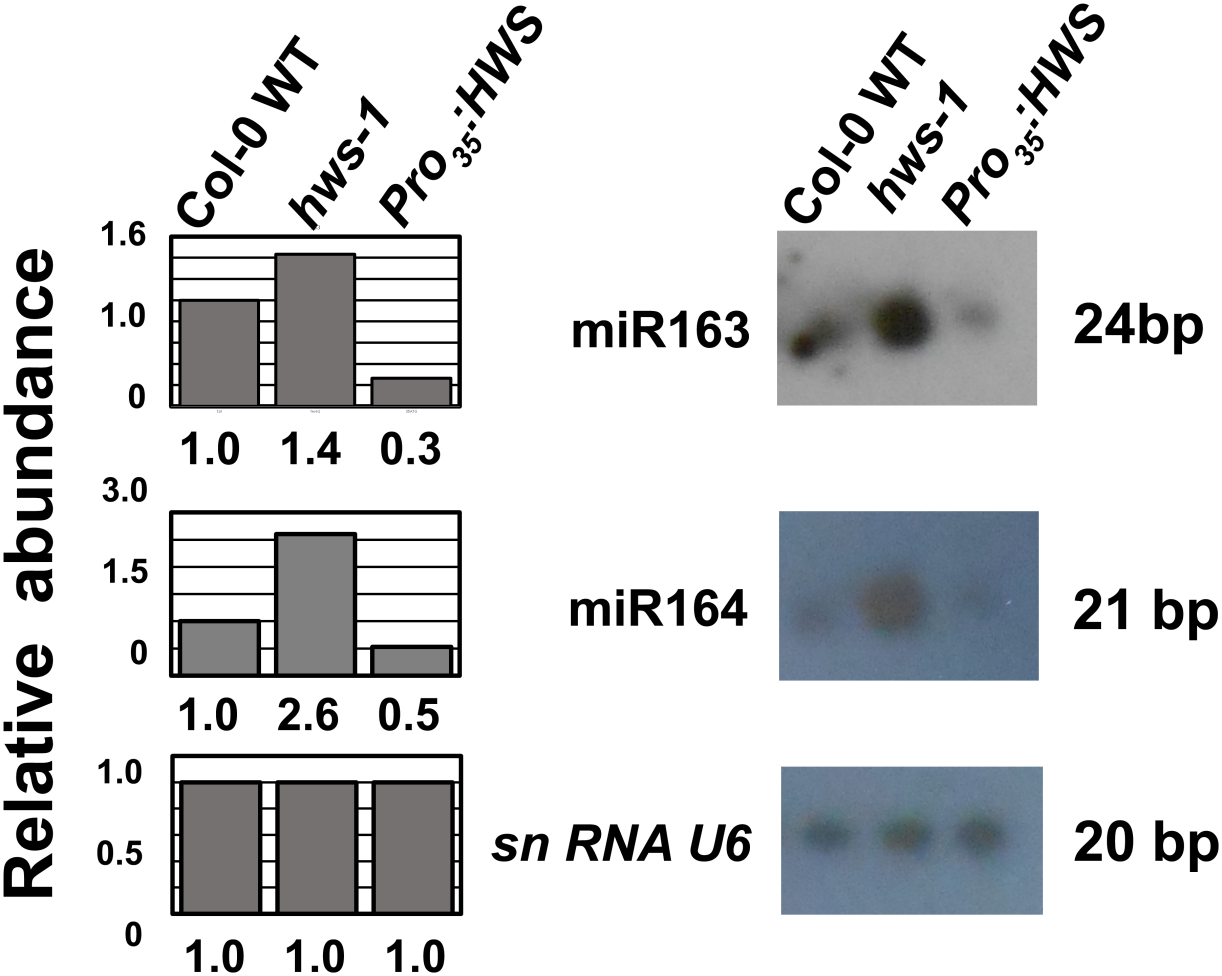
Analysis of mature miRNA accumulation. Northern analyses in a mix of young buds and flowers (up to stage12, [56]) in Col-0 wild type (WT), *hws-1* and *Pro*_*35*_:*HWS* using probes for miR163, miR164, and *snRNA U6* as internal control. Graphs to the left of the miRNA blots indicate the relative abundance of miRNAs compared to the Col-0.

To further address this conclusion, we evaluated the levels of mature miRNAs from MIR163 and MIR164 in developing flower buds, up to stage12 [56]. Compared to the Col-0, significant over-accumulation of miR163 and miR164 was observed in the *hws-1* mutant, while reduction was observed in the *Pro*_*35*_:*HWS* line. (Fig 3). These results support our hypothesis that *HWS* regulates levels of miRNAs in flowers, and likely in other tissues where the *HWS* gene is expressed.

The HWS protein contains an F-box and a Kelch-2 repeat in its C-terminus [15]. F-box proteins are important elements of the E3 SCF complex (from SKP1, Culling and F-box) that catalyse the ubiquitination of proteins to be degraded by the proteasome [62]. It is therefore likely that HWS forms a part of an SCF complex and identifies for targeted degradation protein(s) that are in the miRNA pathway. We performed a yeast-two hybrid screen using a cDNA library generated from stamen tissue from Arabidopsis flowers. A total of 1,280,000 clones were screened. From these, 66 histidine positive colonies were isolated. X-gal assays showed that among the 66 histidine positive colonies, 56 were positive for X- gal. From the 56 X-gal positive clones, 55 contained *Arabidopsis* SKP1 protein; among which, 36 contained only SKP1; 10 contained both SKP1 and PRXR1 (a protein involved in catabolism of hydrogen peroxide), and 9 contained SKP1 and FLA3 (Fasciclin-like arabinogalactan protein 3 precursor). One of the clones contained only SKP4. However, independent X-gal assays could only confirm the interactions between HWS and SKP1 or SKP4, suggesting that the isolated clones may not interact directly with HWS or alternatively interaction of HWS with other proteins require the presence of SKP1 (S2 Fig). These results confirm that the F-box protein HWS is part of an SCF complex likely targeting for degradation protein(s) involved in the miRNA pathway.

### *hws-1* and *hst* mutants reveal epistatic interactions and independent roles of *HWS* and *HST* during plant development

Previously, it was reported that mutation of *HST* induces pleiotropic effects during plant development, which include curling of leaf blades, reduction of leaf numbers, faster production of abaxial trichomes, reduction of leaf, sepals and petals size, laterally expanded stigma, inflorescence phyllotaxy defects and reduced fertility [58, 63–64]. We show here that mutations in *HST* are able to suppress the sepal fusion of *hws-1*.

**Fig 4:**
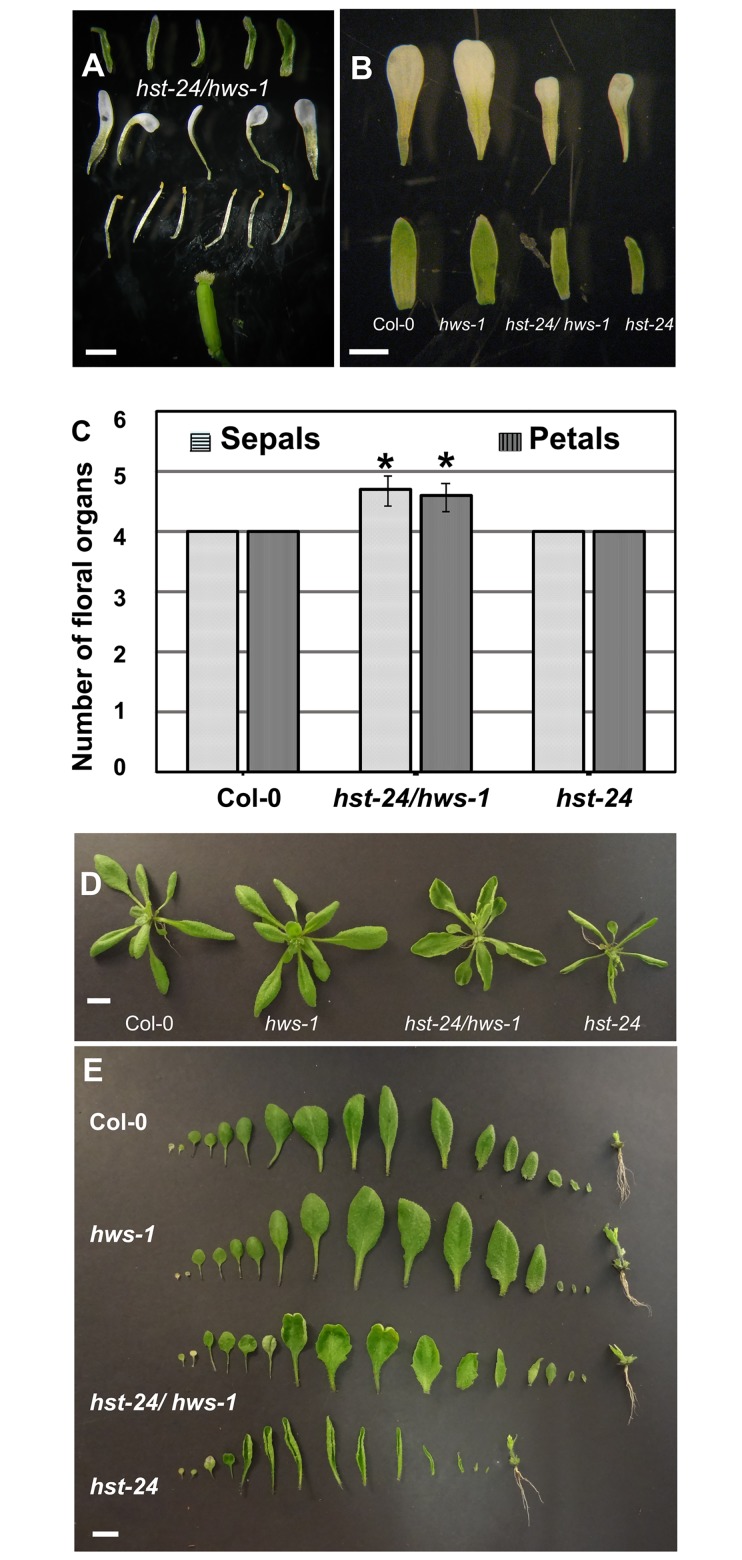
Phenotypic characterisation of *hst-24*. (**A**) Dissected flower from developmental stage 15a from *hst-24/hws-1*. (**B**) Comparative analyses of sepal and petal sizes from flowers (stage 15a) of Col-0, *hws-1*, *hst-24/hws-1* and *hst-24*. (**C**). Twenty-five flowers from six plants of Col-0, *hst-24/hws-1* and *hst-24* were dissected and their sepals and petals quantified and statistically analysed by regression analyses using generalized linear models. Stars indicate a significant difference in the mean at P≤0.001 n = 450. Bars indicate SD. (**D**) Rosettes, and (**E**) Dissected leaves from 22-day-old plants from Col-0, *hws-1*, *hst-24/hws-1* and *hst-24*. Bars in A, B = 1mm; and in D, E = 1 cm.

To understand the biological role of HWS-HST interaction and its role in nuclear export, we addressed if HWS also affects the phenotypic variations associated with *hst* mutants, we performed phenotypic analyses in simple and double mutant lines *hws-1*, *hst-1* and *hst-24/hws-1*, *hst-23/hws-1*. Indeed, a reciprocal complementation of *hst* phenotypes by mutating *HWS* was observed when analysing *hst-23/hws-1* and *hst-24/hws-1* double mutants. Mutation of HWS (*hws-1*) was able to suppress phenotypes associated with *hst* mutations, such as the curling up of the leaf blades, the reduction of leaf numbers, the reduction of silique dimensions and fertility, the reduction of the expansion of stigmas and the disorientation of petals (Figs 1Q, 1R, 1S, 1T, 1U, 1V, 1W, 1X, 4D and 4E and S1 Fig). These results are in agreement with the data above and corroborate that HWS acts in the miRNA pathway.

However, mutation of *HWS* could not supress other phenotypes associated with the *hst* mutation. Sepals and petals from *hst-24* were reduced in size compared to that of Col-0 and*hws-1* (Fig 4B). Sepals and petals of double mutant *hst-24/hws-1* were comparable in size to the ones from the *hst-24* single mutant demonstrating that loss of function of *HWS* was not able to supress the reduced petal size phenotype associated with the *hst* mutation (Fig 4B). This observation suggests that *HST* must perform other functions independently of *HWS*.

Phenotypic analyses of flower organ number in *hst-24* mutant showed the characteristic four sepals and four petals (Fig 4C and Table 1). However, a statistically significant (p<0.0001) increase of sepals and petals number of 10% was observed in the double mutant *hst-24/hws-1* (Figs 1E, 4A, 4B and 4C and Table 1). Interestingly, the increments were only observed in the first ten flowers of each plant analysed, the subsequent fifteen flowers analysed displayed floral organ number comparable to the wild type. Approximately 58% of the flowers had an increase of both sepals and petals within a single flower. Taken together these data suggest that HWS interacts with HST in the miRNA pathway to control some biological functions, but must also act in an independent pathway to control others.

**Table 1:** Mean of sepal and petal numbers in Col-0, *hws-1*, *hst-24/hws-1* and *hst-24* in Col-0 from the first 25 flowers of the inflorescences, (flowers n = 200).

GENOTYPE	Sepals		Petals	
	Mean±SD		Mean±SD	
	Organ number	(Min-Max)	Organ number	(Min-Max)
**Col-0**	4±0	(4–4)	4±0	(4–4)
***hws-1***	4±0	(4–4)	4.1±0.31	(3–5)
***hws-1/hst-24***	4.4±0.5	(4–5)	4.4±0.7	(4–6)
***hst-24***	4±0	(4–4)	4±0	(4–4)

## Discussion

Although plenty of knowledge has been generated since the discovery of the first miRNAs in 1993 [65–67], the complexity of mechanisms regulating their biogenesis, expression and mode of action is not fully elucidated. Here we demonstrate a role for *HWS* in the miRNA pathway. Our first line of evidence comes from the isolation of two new *HST* alleles, *hst-23* and *hst-24*, from a screening of EMS *hws-1* mutant suppressor lines. These alleles were able to suppress the sepal fusion phenotype from *hws-1*. HST has been implicated in the export of an unidentified component of the miRNA pathway, miRNA biogenesis or miRNA function [48]. Our second line of evidence comes from our genetic crosses between *hws-1* or *hws-2* and *ddl-2*, *se-1*, *hyl-1*, *dcl1-9*, *hen1-5*, *hst-1* and *ago1-37* mutants from known genes regulating the biogenesis and function of miRNAs, that show suppression of the sepal fusion from *hws-1*, demonstrating that *HWS* has a role in biogenesis, stability and/or function of miRNA in addition to their transport involving HST. Interestingly, there was a noticeable reciprocal suppression of phenotypes between the *hws* and *ddl-2*, *dcl1-9* and *hen1-5* mutants in floral development, fertility and flower morphology, suggesting epistatic interactions. Suppression of phenotypes towards the end of flower production was less apparent, suggesting that the regulatory mechanisms becomes altered in a spatiotemporal way, or that HWS is targeting for degradation a yet to be identified protein that regulates genes of the miRNA pathway in a spatiotemporal fashion upstream of the miRNA biogenesis process. Alternatively, a compensatory mechanism to regulate microRNA biogenesis could be present; in agreement with this hypothesis, it has been previously demonstrated that such mechanisms exist to compensate cell number and associated organ sizes defects in plants [68]. Our third line of evidence comes from our Northern blot analyses where differential accumulation of mature miR163 and miR164 in floral tissues in the *hws-1* mutant and the *Pro*_*35*_:*HWS* line were observed, suggesting that during development a differential regulation of mature miRNAs is required, and this is achieved by a pathway implicating HWS. It is known that miR163 negatively regulates mRNA levels of *PMXT1*, a member of the S-adenosyl-Met dependent carboxyl methyltransferase family, to modulate seed germination, seedling de-etiolation and root architecture in response to light [69]. While miR164 negatively regulates mRNA levels of *CUC1* and *CUC2* genes to modulate boundary formation in flowers [14, 17–18]. Our Northern blot results provide further evidence for a role of HWS in miRNA pathway and suggest that the sepal fusion phenotype observed in *hws-1* maybe due to the over accumulation of miR164 which in turn modulates mRNA levels of *CUC1*, and *CUC2*.

Our data point to the hypothesis that putative target proteins of HWS, act upstream of the miRNA biogenesis pathway, or affect miRNA stability or function, or a combination of all of these. The HWS protein holds an F-box and a Kelch-2 repeat in its C-terminus [15]. It is likely that the interaction between HWS and its targets involves the Kelch-2 repeat. In agreement with this proposal, in our yeast-two-hybrid experiments we were able to demonstrate that HWS interacts with ASK1 and ASK4, two proteins that are part of the SCF complex, supporting the idea that HWS role in the miRNA pathway may be by targeting proteins for degradation through the SCF complex.

Although these targets remain to be identified, putative candidates could be PROTEIN PHOSPHATASE 4 (PP4), SUPPRESOR OF MEK1 (SMEK1) [42], REGULATOR OF CBF GENE EXPRESSION (RCF3) or C-TERMINAL DOMAIN PHOSPHATASE-LIKE1 AND 2 (CPL1 and CPL2), that are known to be involved in controlling the phosphorylation status of HYL-1 to promote miRNA biogenesis [43]. Alternatively, the CAP-BINDING PROTEINS 20 and 80 (CBP20 and CBP80, also known as ABH1), important proteins during the biogenesis of miRNAs and ta-siRNA biogenesis [70]. It has been demonstrated that ABH1 (CBP80) is also able to suppress the *hws-1* sepal fusion phenotype [71]. Therefore, CPB20 and CBP80 are strong candidates for targeted degradation through HWS. In the literature, some redundancy and cross-talk between known pathways generating miRNAs, ta-siRNAs and siRNAs, and other pathways that remain to be discovered, has been reported [72]. The role of HWS in the regulatory events during ta-siRNAs and siRNAs biogenesis pathways, among others, remains to be elucidated. Testing interactions of these proteins will shed light of the putative role of HWS in controlling the phosphorylation status of key players in the miRNA pathway.

It has been suggested that the AUXIN SIGNALING F-BOX 2 (*AFB2*) gene is post-transcriptionally negatively regulated by miR393, and a regulatory mechanism where miRNAs prevent undesired expression of genes involved in miRNA production has been proposed [73]. An alternative to this suggestion comes from the finding of numerous siRNAs in the proximity of the *MIR393* target site for the F-boxes *TIR1*, *AFB2*, and *AFB3* genes [74]. [74] suggested that the regulation of their transcripts occurs via siRNAs rather than *MIR393*. Further experiments will establish if this regulatory mechanism holds true for *HWS*.

We revealed that the *hws-1* is able to suppress the curling up of leaf blades, reduction of leaf numbers, reduction in leaf size, expansion of stigma, petal orientation, and reduced fertility phenotypes characteristic of *hst* mutants [58, 63–64]. However, HWS and HST seem to also have independent roles as the *hws* mutation could not supress some phenotypes associated with the *hst* knockout. Moreover, the double mutant *hst/hws* exhibited increased sepals and petal number in the first ten formed flowers, a phenotype not seen in the *hst-24* or *hws-1* single mutants. The underlying mechanisms of the increased number of sepals and petals in the double mutant remain unknown. It has been reported that *HST* affects bolting and floral maturation timing [63], but there are no reports of HST affecting floral organ numbers. These findings suggest epistatic interactions between *HWS* and *HST* to fine tune development in plants, in a spatiotemporal way, in addition to independent roles for HWS and HST in plant development.

Previous findings point to the fact that genes involved in the miRNA pathway must have other roles in addition to miRNA biogenesis, transport or function. For example, *ddl* mutants have more severe morphological phenotypes than these of the *dcl1-9* mutants; but the miRNA levels are reduced in the *dcl1-9* compared to the *ddl* mutants [35]. Moreover, it has been demonstrated that DDL regulates plant immunity by poly(ADP‐ribosyl)ation (PARylation) of proteins; and regulates plant development via the miRNA biogenesis pathway [75]. Another example is illustrated by CBP20 and CBP 80. It has been demonstrated that in addition to their role in miRNA biogenesis these proteins also act during the formation of a heterodimeric complex that binds the 5’ cap structure of a newly formed mRNA by Pol II, aid in the pre-miRNA splicing and act during polyadenylation and during the export of RNA out of the nucleus [70, 76–80]. Therefore, it is likely that both *HWS* and *HST* have additional roles to that of miRNA pathway.

Our data shed light on the complexity of mechanisms regulating miRNA pathway, and place *HWS* as a new regulator in this pathway. In support of our findings, [71] have proposed HWS as a regulator of miRNA function in their screening studies for negative regulators of *MIR156* activity.

Due to the impact on development that HWS exerts, this research is relevant for identifying novel strategies to generate more productive and resilient crops. As support to this, recently we showed that a mutant from the *ERECTA PANICLE3*, the *HWS* rice orthologue gene in rice, has decreased photosynthesis due to reduced stomatal conductance and attenuated guard cell development [81]. Moreover, [82], demonstrated that Arabidopsis mutants and a knock down line of *OsFBK1*, a second *HWS* rice orthologue gene, germinate better and have root systems that are more robust on exposure to ABA than wild type, important for drought tolerance.

## Supporting information

S1 Fig Phenotypic characteristics of *hst-23*. (**A**) F1 progeny and (**B**) flower from a cross between *shs-2/hws-1 and shs-3/hws-1* demonstrating that *shs-2* and *shs-3* are allelic. (**C**) Dissected rosette and cauline leaves from 22-day-old plants from: Col-0, *hst-23/hws-1*, *hst-23*, *hst-1*x*hws-1* and *hst-1*. Bars in A, C = 1 cm, in B = 1mm.
(TIF)

S2 Fig Yeast-two-hybrid interactions. **(A-E)** Sixty-six histidine positive clones, identified from a screening using a stamen cDNA library from Arabidopsis flowers, were analysed for β-galactosidase activity. **(F)** Individual clones tested for protein-protein interactions: (1) SKP1, (2) SKP4, (3) PRXR1 and (4) FLA3. Positive clones are shown in blue. Ac-Ec, are positive controls where A is the weakest control and E is the strongest control.
(TIF)

S1 Table Primers and probes used in this study. Marker, sequencing, screening, yeast-two-hybrid primers and probes used in Northern blots are included.
(DOCX)
