# Supplementary figures and images for: The Arabidopsis thaliana F-box gene HAWAIIAN SKIRT is a new player in the microRNA pathway
Source: PLoS One. 2017 Dec 15;12(12):e0189788. doi: 10.1371/journal.pone.0189788 (PMC5731758; doi:10.1371/journal.pone.0189788)

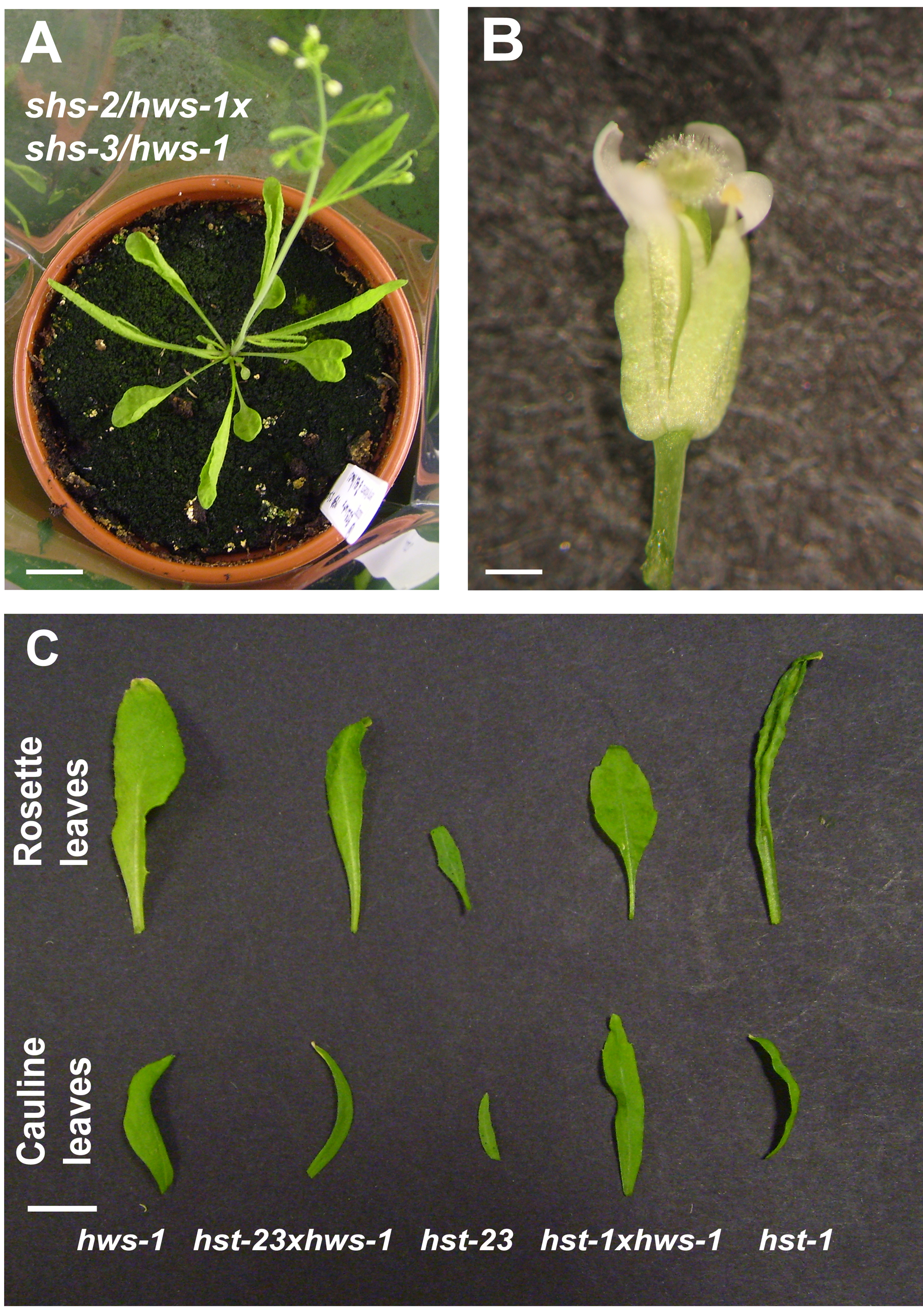

Supplement: S1 Fig — (A) F1 progeny and (B) flower from a cross between shs-2/hws-1 and shs-3/hws-1 demonstrating that shs-2 and shs-3 are allelic. (C) Dissected rosette and cauline leaves from 22-day-old plants from: Col-0, hst-23/hws-1, hst-23, hst-1xhws-1 and hst-1. Bars in A, C = 1 cm, in B = 1mm. (TIF) [file pone.0189788.s001.tif]

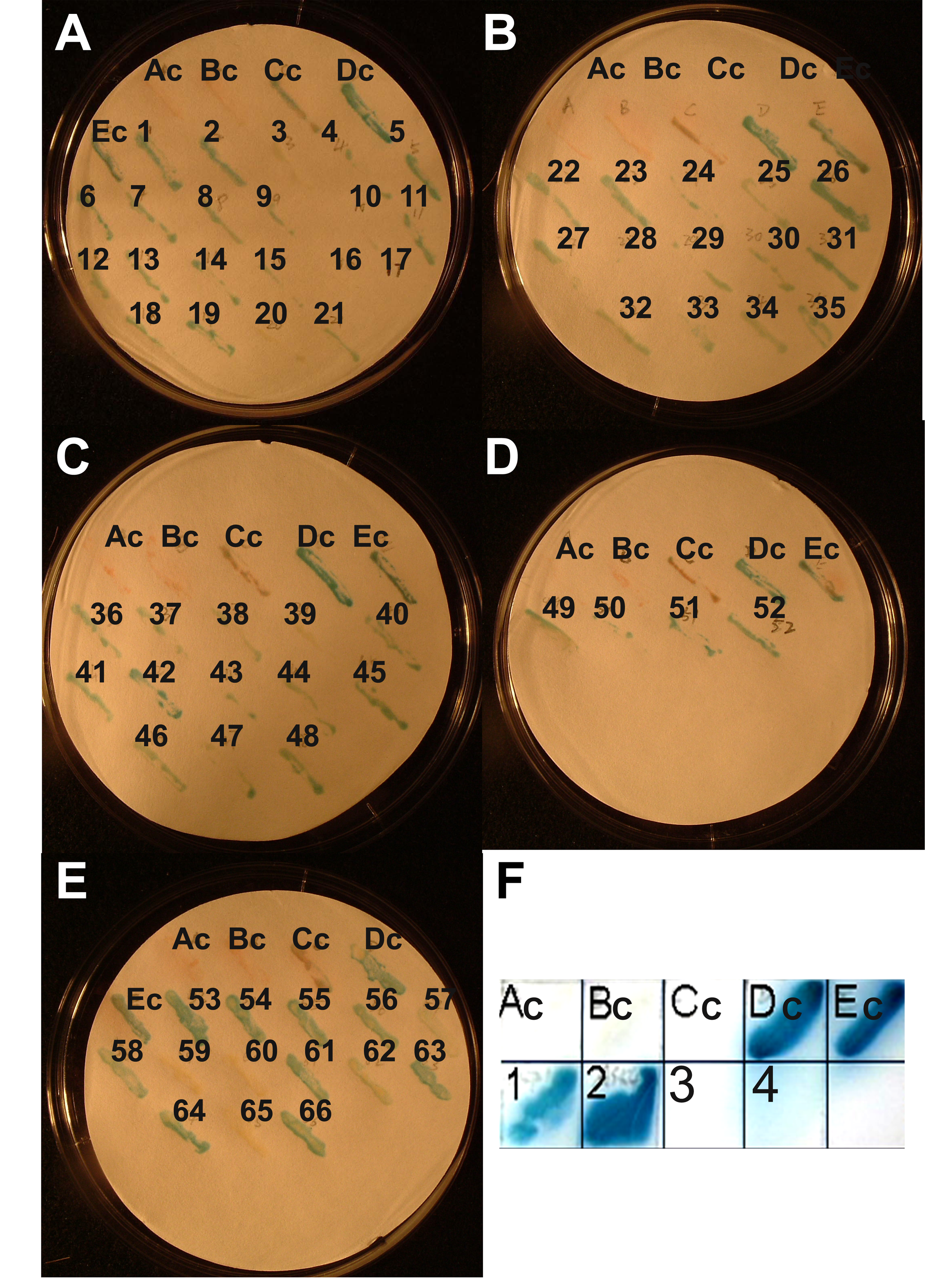

Supplement: S2 Fig — (A-E) Sixty-six histidine positive clones, identified from a screening using a stamen cDNA library from Arabidopsis flowers, were analysed for β-galactosidase activity. (F) Individual clones tested for protein-protein interactions: (1) SKP1, (2) SKP4, (3) PRXR1 and (4) FLA3. Positive clones are shown in blue. Ac-Ec, are positive controls where A is the weakest control and E is the strongest control. (TIF) [file pone.0189788.s002.tif]
