# Supplementary material for: The Arabidopsis thaliana F-box gene HAWAIIAN SKIRT is a new player in the microRNA pathway
Source: PLoS One. 2017 Dec 15;12(12):e0189788. doi: 10.1371/journal.pone.0189788 (PMC5731758; doi:10.1371/journal.pone.0189788)
Supplement: S1 Table — Marker, sequencing, screening, yeast-two-hybrid primers and probes used in Northern blots are included. (DOCX) [file pone.0189788.s003.docx]

**S1Table S. Primers and probes used in this study.**

Marker, sequencing, screening, yeast-two-hybrid primers and probes used in Northern blots are included.

| **Mapping primers for *shs-2*:** | | | |
| --- | --- | --- | --- |
| **Marker name** | **Position on chromosome 3 (bp)** | **Primers (F, forward; R, reverse)** | **PCR product in bp (Col/L*er*)** |
|  |  |  |  |
| CER452410 | 242,121 | F: TTCGTCGACTTCTCTCACACA | (130/104) |
|  |  | R: TTTATTCACCAACAACCCAGA |  |
| CER450479 | 942,679 | F: CACACGTGCAAAGAGAGTG | (184/166) |
|  |  | R: GTTTCAGACGCAATGTACGC |  |
| 474020 | 1,161,859 | F: TTTAGGGTTCTTGATTGGTG | (86/78) |
|  |  | R: CAGCTATCCTTAAAGATCTG |  |
| CER461407 | 1,354,319 | F: GGAATTATTACTATTTGCAAG | (112/101) |
|  |  | R: ATTCACAAGCTAGAGAAAAC |  |
| 471430 | 1,413,773 | F: TCCTTCAAGATTTCTCAACC | (111/108) |
|  |  | R: CTCTTGTTGGTATCTCTCTG |  |
| 470580 | 1,421,838 | F: AGAATGCGACAATGAGAAGC | (85/81) |
|  |  | R: TTTCTTTATAGTTCCCACTG |  |
| 470582 | 1,456,284 | F: TTTTCTCTCAATGTAGTGTG | (122/119) |
|  |  | R: TATCTAGTTGTTTCTTGTGG |  |
| 470584 | 1,479,253 | F: GTCAATTTCCATCCAGAAGG | (97/92) |
|  |  | R: GCATGTAATATAACGGAGCG |  |
| 470579 | 1,540,131 | F: TTATAGGTTAGCGATTGAAG | (146/133) |
|  |  | R: ATCAATTACACACAGTGAAG |  |
| 473863 | 1,582,917 | F: GTCACCACATTAATTCCAAGA | (205/140) |
|  |  | R: TGGTAACACCCTCTTTCTCCA |  |
| 470677 | 2,299,587 | F: CCCAAATCCACCGAACATAA | (153/115) |
|  |  | R: CCAAAAACGCCAACTTCTT |  |
| 470642 | 2,706,391 | F: ATCTGACGTGGACGGAATCT | (127/102) |
|  |  | R: GAGTGTAGTGGCCGTTGGAT |  |
| **Sequencing primers for *hst-23* and *hst-24:*** | | | |
| **Primer name:** | | **Sequence (5’→3’)** | |
| HSTfor | | ATGGAAGATAGCAACTCCACGGCAAG | |
| HSTrev | | CACAGGCAACGATTGAGTAAGTCC | |
| HST1for | | GTGGGTTATATGTTCTTTATTTGG | |
| HST1rev | | CTCAGCTACAAGGGCAGCAGACTG | |
| HST2for | | CATTTGGTTAGACTACGATGGGACG | |
| HST2rev | | CACGTAATGCATAATGAACTTCAG | |
| HST3for | | GATGACATCTCGAGCGCTATACTGG | |
| HST3rev | | GGTGCAGAAGCACTCCCAGCCATG | |
| HST4for | | GAACTAAGGAAGGACAAGCCGAGGC | |
| HST4rev | | CCAATTGACCGATTGTTTAAATACTG | |
| HST5for | | GAACTAAGGAAGGACAAGCCGAGGC | |
| HST6for | | CTCACCTCTCTTCCACACGTTGTG | |
| HST7for | | GATGGAGAAGCGACCACCAAAGTC | |
| HST8for | | CATTTGGTTAGACTACGATGGGACG | |
| **Detection primers for *hst-23* mutants:** | | | |
| 23.1revmodwt | | GCCAGGCGGAGCTGAGAGCTTACTG | |
| 23.1revmodmut | | GCCAGGCGGAGCTGAGAGCTTACTA | |
| 23.1formdwt | | CTGCACCCATTGTTTATACACCGTC | |
| 23.1formodmut | | CTGCACCCATTGTTTATACACCGTT | |
| 23.1revscr | | CCCACCATAGAGTTGGATCTGAACG | |
| **Detection primers for *hst-24* mutants:** | | | |
| HSTForwardM | | CTTTTCATCTTGTGAAAAAAGAATA | |
| HSTForwardWT | | CTTTTCATCTTGTGAAAAAAGAATG | |
| **Detection primers for mutants of the miRNA pathway:** | | | |
| AGO1-37forwt | | ACTTGATGCCATCCGCGAGG | |
| AGO1-37formut | | TGAACTTGATGCCATCCGCTAGA | |
| AGO1-37rev | | GCAGATTTTAGAGTCCACAACAGG | |
| FORDCL1-9 | | GCATATCAACAACGGTAATGCG | |
| REVDCL1-9 | | CATCGGTCCATCCTCTATCG | |
| DDLSLKFOR | | CGACCCTGACTCCAATAATGGCTCC | |
| DDLSLKREV | | CATTCTCGCGACTGAATCTTCCTCG | |
| DDLWSFOR | | GACGTAAGAGGCTACGTATGTTCGCT | |
| DDLWSREV | | CTTGCTTCCCCATCATACCATCTGGT | |
| HEN1FOR | | CAGAAGTTACTCAGATGACTGTGG | |
| HEN1REV | | GGTGTAGACCGCTGGAGAATTGTG | |
| HSTFORWT | | TCTCCAGCTCCTCTTCATGT | |
| HSTFORMUT | | TCTCCAGCTCCTCTTCATGA | |
| HSTREV | | CACGTAATGCATAATGAACTTCAG | |
| SE11extronFor | | CTGTTGTCTCCGGCCTTT | |
| SEcDNArev | | AGCCCTGTCTTGTCTACA | |
| HYLMutfor | | CCTGCTCAGGTGTTTCCAATTGC | |
| HYL1FOR | | TCGTGAAAATGACCTCCACTGATG | |
| HYL1REV | | GGTTGTGAAACACATTGGCTTAGC | |
| **Y2H Primers:** | | | |
| HWS_attB1 | | GGGGACAAGTTTGTACAAAAAAGCAGGCTT  GAGAATGGAAGCAGAAACG | |
| HWS_attB2 | | GGGGACCACTTTGTACAAGAAAGCTGGGTT  CTTCATTGCAACTAAGGA | |
| **Probes for Northern blots:** | |  | |
| MIR163 | | ATCGAAGTTCCAAGTCCTCTTCAA  TTTTCCTGTCTC | |
| MIR164 | | TGGAGAAGCAGGGCACGTGCATT  TTCCTGTCTC | |
| SnRNA U6 | | TCATCCTTGCGCAGGGGCCATTTTCCTGTCTC | |
